# Bilateral jaws involvement of Burkitt’s lymphoma in a pediatric patient

**DOI:** 10.4317/jced.57740

**Published:** 2021-03-01

**Authors:** Silas-Antonio-Juvencio de Freitas Filho, Ludimila-Lemes Moura, Mário-César de Souza, Cassia-Maria-Fischer Rubira, Denise-Tostes Oliveira

**Affiliations:** 1 Department of Surgery, Stomatology, Pathology and Radiology, Bauru School of Dentistry, University of São Paulo, Bauru, São Paulo, Brazil; 2 Department of Dentistry, Northern State University of Paraná, Jacarezinho Unit, Paraná, Brazil

## Abstract

A case of Burkitt’s lymphoma with bilateral jaws involvement in a 5-year-old boy is reported discussing the dentist’s role in the diagnosis and management of this disease. The initial clinical diagnosis established of maxillary swelling causing trismus was a dentoalveolar abscess. The incisional biopsy was performed and histopathological analysis, including immunohistochemistry, confirmed the Burkitt’s lymphoma. The patient underwent treatment and remains free of the disease for 36 months of follow-up. The occurrence of intraoral bilateral jaws involvement of Burkitt’s lymphoma in child is unusual and its accurate diagnosis avoids complications in the patient’s treatment.

** Key words:**Burkitt’s lymphoma, Non-Hodgkin’s lymphoma, Oral cavity, Maxilla, Child.

## Introduction

In 1958, Denis Parsons Burkitt, an Irish surgeon, published the first study about mandibular tumors that affected African children, until then called “small round cell sarcoma” (1). Three years later, Burkitt together with Gregory O’Conor defined the malignancy as a lymphoma (1).

Currently, Burkitt’s lymphoma is known to be a B-cell malignancy and classified as a highly aggressive non-Hodgkin’s lymphoma (2-4). Three clinical subtypes of this malignancy are recognized: 1) endemic, associated with the Epstein-Barr virus; 2) sporadic; 3) associated with immunodeficiency, mainly HIV infection (2,4-6). Burkitt’s lymphoma involving the gnathic bones has been more common in the endemic subtype (2,4).

Here we intend to report a case of Burkitt’s lymphoma in a 5-year-old child, affecting bilaterally jaws, with significant maxillofacial asymmetry, pain, trismus, and with an initial clinical diagnosis of a dentoalveolar abscess. The role of dentist in the diagnosis and management of Burkitt’s lymphoma in pediatric patient is discussed.

## Case Report

A 5-year-old boy attended to the Buccomaxillofacial Surgery and Traumatology Service, accompanied by his mother, complaining of bilateral mandibular swelling, trismus, pain on the left side of the face and headache.

**Figure 1:**
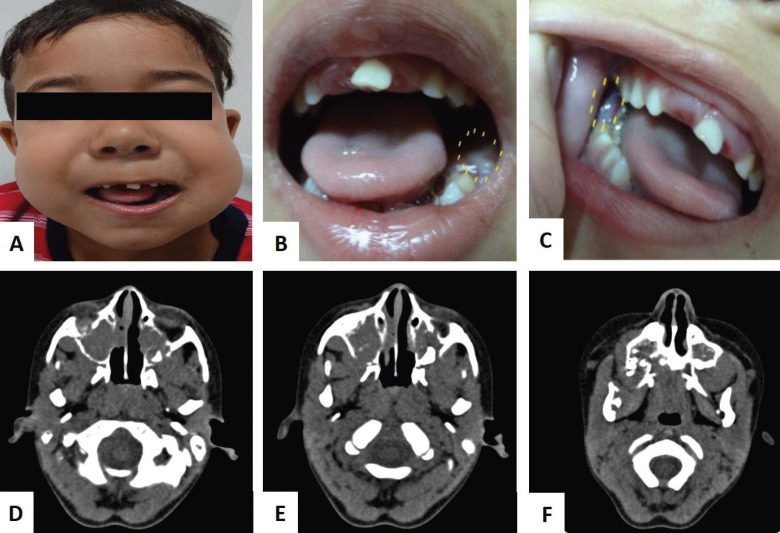
(A-C) Initial clinical facial asymmetry. Frontal view of the face prior to the intraoral incisional biopsy. Intra-oral maxillary jaws swelling causing limited mouth opening and ulceration in the left alveolar ridge. (D-F) Axial CT scan showing mass invasion causing destruction of orbits floor, both maxillary sinus and maxilla.

During anamnesis, his mother reported that the patient had been previously examined by a dentist and the initial clinical diagnosis of dentoalveolar abscess was established, but there was no surgical intervention due to rapid contralateral swelling (right side). The extraoral examination revealed facial asymmetry due a bilateral volumetric increase (Fig. 1A). The intraoral examination was not performed properly, as the child did not cooperate due to painful symptoms and trismus. However, ulceration in the left alveolar ridge (Fig. 1B), swelling with color change in the right alveolar ridge (Fig. 1C) and tooth mobility of the 75 were observed. A contrast-enhanced CT scan of the maxillo-facial revealed a solid tissue invading both sides of maxilla and the maxillary sinus and partially the floor of orbitals (Fig. 1D-F). Serum lactate dehydrogenase levels were increased by two times compared to reference values.

**Figure 2:**
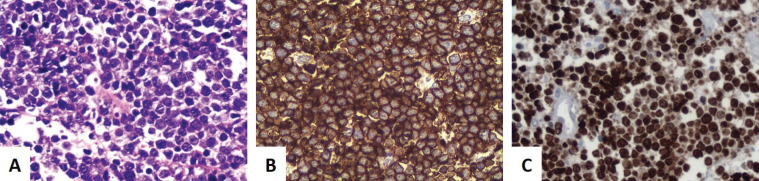
Histopathological and immunohistochemical analyses. (A) Representative photomicrograph demonstrating numerous monomorphic neoplastic lymphoid cells of intermediate size, sparse cytoplasm, rounded nuclei and peripheral nucleoli, in addition to vacuolated macrophages (hematoxylin and eosin stain, 40x magnification). (B) CD20 positivity in neoplastic cells with finely granular marking pattern in the cytoplasm and strong in the plasma membrane region (immunoperoxidase stain, 20x magnification). (C) Positivity for Ki-67 in almost 100% of the neoplastic cell nuclei (immunoperoxidase stain, 20x magnification).

Under general anesthesia and with nasotracheal intubation, an intraoral incisional biopsy was performed in the region of the cul-de-sac and alveolar ridge of the right maxilla. Histopathological analysis revealed atypical monomorphic lymphoid proliferation with the involvement of the adjacent bone tissue (Fig. 2A). Immunohistochemical analysis using the following primary antibodies CD20, CD10, BCL6, Ki-67, BCL2, CD23, CD3, CD5 and Cyclin D1 was performed as shown in Table 1 and Fig. 2B-C. Based on histopathological and immunohistochemistry analyses the diagnosis of Burkitt’s lymphoma was established. The serological test was negative for HIV and the boy did not have immunodeficiency in his medical history. EBV status and C-MYC gene rearrangement have not been investigated.

**Table 1:**
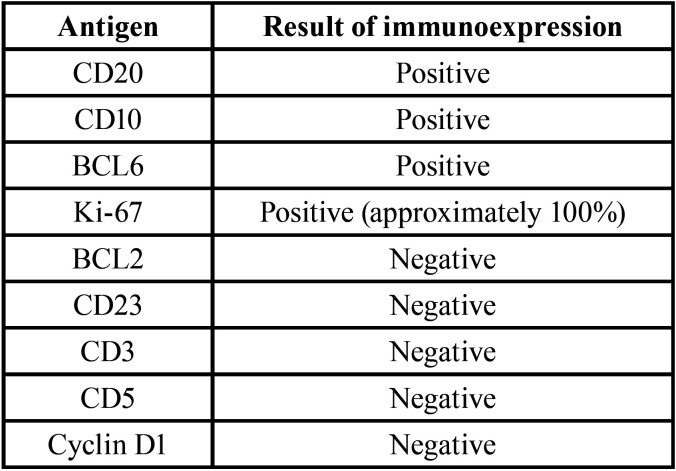
Panel of immunohistochemical analysis of the present case.

**Figure 3:**
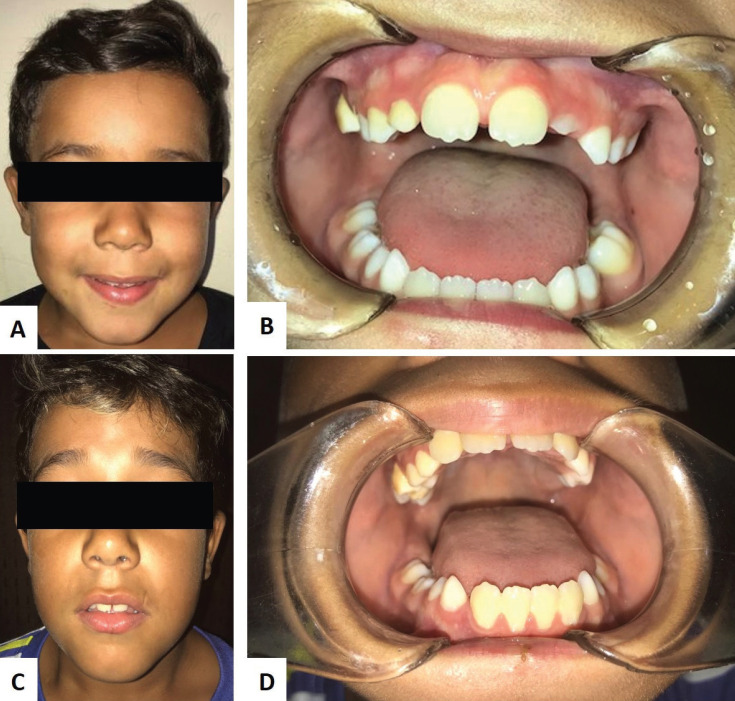
(A-B) and (C-D) After 12 and 24 months of treatment, the patient shows complete remission of the Burkitt’s lymphoma.

The patient underwent treatment in a Specialized Oncology Center following the protocol of the Brazilian Society of Pediatric Oncology, with cytoreduction therapy of conventional chemotherapy and intrathecal chemotherapy. Clinical images after 12 and 24 months of treatment are shown in Figure 3. After 36 months of follow-up, no signs of tumor recurrence were observed.

## Discussion

The three main groups of oral and maxillofacial lesions that often affect children are represented by pathologies of salivary glands, reactive lesions and odontogenic cysts (7). Malignant tumors in children are extremely rare, accounting for less than 1% of all lesions (8,9), and Burkitt’s lymphoma has been the most common of them (7,9). Although girls are more affected by maxillofacial lesions (7), boys have been more affected by Burkitt’s lymphoma (2,10). Our case shows some typical characteristics of this neoplasm, such as sex, age and unusual features such as bilateral jaws involvement.

In the present case, the swelling was the main clinical aspect of the disease, in addition to the presence of a small area of ulceration in the alveolar ridge. This clinical observation is in line with the recent study by Rodrigues-Fernandes *et al*. (2), which show that maxillofacial swelling is the most frequent clinical sign in children with this malignancy. Although bilateral involvement of Burkitt’s lymphoma is uncommon (11), as shown in the present case, this aspect was decisive in eliminating the hypothesis of an infectious or inflammatory lesion.

Maxillary involvement is more common in the endemic subtype of Burkitt’s lymphoma and rare in sporadic cases (4). Our patient was not investigated for the presence of HBV, and a definitive classification is not possible. Also, more 89% of pediatric patients reviewed by Rodrigues-Fernandes *et al.* (2) do not have information on the identification of EBV in these tumors. Additionally, the incidence of Burkitt’s lymphoma cases has been considered intermediate (between endemic and sporadic) in different regions of the world, including South America (2).

The main symptoms, such as pain and altered sensitivity, can make clinical diagnosis challenging. The signs and symptoms of Burkitt’s lymphoma in the child’s maxillofacial region may be mistaken for an acute inflammatory process (dentoalveolar abscess or periapical lesion), fibro-osseous lesion or other neoplasms with aggressive behavior (11). In the present case, a dentoalveolar abscess was initially suspected due to the significant volumetric increase in the left side associated with pain. Although infrequent in children, it was only after the swelling on the right side that an investigation of a neoplastic disease began. In addition, our patient, similar to the case reported by Freitas *et al*. (12), presented several alterations that can be found in other diseases that affect the maxillofacial complex, such as swelling, intraoral tumor mass and tooth mobility.

Despite the intraoral involvement by the neoplasm, it was possible to maintain the child’s oral hygiene. The follow-up between chemotherapy sessions to maintain oral care and reinforcement with the patient and family are conducts that must be adopted as recommended by Padmanabhan *et al.* (13) to avoid or reduce the side effects of treatment.

The destruction of the cortical bone of the maxilla and mandible was an important finding in the imaging examination of the present case. The hypodense tumor mass occupied regions such as peribuccal spaces, maxillary sinuses, nasal floor and partially the infraorbital region. Likewise, the involvement of these regions by Burkitt’s lymphoma has been described in the literature (4,12,13). In general, the lesions have imprecise limits of invasive appearance, are multiple and can coalesce, with loss of alveolar support of the teeth being common (4,13).

The accurate diagnosis of this malignancy is challenging, but it avoids complications in the management and treatment of the patient (3,6). In association with routine morphological analysis, the development of an immunohistochemical panel is essential to obtain the definitive diagnosis (6,14). The antigens with negative results shown in Table 1, ruled out diagnostic hypotheses of mantle cell lymphoma, diffuse large B-cell lymphoma and immunophenotype T lymphomas. On the other hand, the presence of immunoexpression for CD20, CD10, BCL6 and Ki-67 it was essential to establish the safe diagnosis of Burkitt’s lymphoma.

The chemotherapy cycles to treat Burkitt’s lymphoma, as in this case, allow the complete remission of the disease. The chemotherapy treatment adopted in pediatric patients provides a relative survival rate of 90.4% in five years of follow-up (5). Additionally, radiotherapy can be indicated for patients with a potential risk of involvement of the central nervous system or in cases that require rapid decompression of structures of the same system due to the disease’s involvement (4,15).

In summary, the occurrence of maxillary malignancy in children is rare and in conditions of rapid tumor growth, as in the case reported, the dentist must be promptly trained to make decisions based on medical history and clinical and radiographic aspects. The microscopic analysis in association with immunohistochemistry allows for accurate diagnosis, as well as providing adequate management.
